# O06 - The Chelsea, asthma and fresh fruit intake in children (CHAFFINCH) trial – pilot study

**DOI:** 10.1186/2045-7022-4-S1-O6

**Published:** 2014-03-14

**Authors:** Vanessa Garcia-Larsen, Andrew Bush, Robert J Boyle, Seif O Shaheen, John O Warner, Toby Athersuch, Ian Mudway, Peter GJ Burney

**Affiliations:** 1 Respiratory Epidemiology and Public Health Group, National Heart and Lung Institute, Imperial College London, London, United Kingdom; 2 Royal Brompton Hospital and Harefield NHS Foundation Trust, London, United Kingdom; 3 Department of Paediatrics, Imperial College London, London, United Kingdom; 4 Centre for Health Sciences, Blizard Institute, Barts and The London School of Medicine and Dentistry, London, United Kingdom; 5 School of Public Health, Imperial College London, London, United Kingdom; 6 Environmental Research Group, King’s College London, London, United Kingdom

## Background

Observational evidence suggests that intake of fruits, and of dietary antioxidants would be protective against symptoms and severity of asthma in children. Intervention studies with single or combined antioxidant supplements have so far been disappointing. To date, there have been no interventions using fresh fruit on asthmatic children. We plan to carry out a 3 month randomised controlled trial (RCT) in 360 children, to establish whether increasing fruit intake could be useful in the management of childhood asthma.

## Objective of the Pilot

To assess recruitment strategy, follow-up, tolerance to respiratory and biomarker tests, and fruit intake compliance (FIC).

## Methods

32 children aged 6 to 10 years old with mild to severe asthma were recruited and randomly allocated to one of the following fruit groups daily, for a month, in addition to their usual diet: 1) an apple, 2) a banana, 3) an apple and a banana, 4) control. Quality of life, lung function, and inflammation were measured with the Juniper Asthma Quality of Life Questionnaire (JAQoLQ), pre- and post- bronchodilator spirometry, and exhaled nitric oxide (eNO), respectively, at baseline and at follow-up. FIC was measured through a dietary questionnaire (DQ) and sticker charts. Urinary metabolomic analyses (UMA) were carried out to identify specific metabolites of fruit intake.

## Results

28 children (88%) completed all the tests at baseline and at follow-up. All children were able to perform satisfactory respiratory and eNO tests. Levels of eNO were 18% lower in groups 2 and 3 after the intervention. DQ and sticker charts were the best FIC methods, with a 96% adherence. UMA showed higher levels of polyphenol antioxidants after ingestion of fruits.

## Conclusion

A RCT with fresh fruit is a feasible intervention in small children with various levels of asthma.

